# Tumour necrosis factor‐α promotes BMHSC differentiation by increasing P2X7 receptor in oestrogen‐deficient osteoporosis

**DOI:** 10.1111/jcmm.16048

**Published:** 2020-11-09

**Authors:** Jiajia Lu, Zhibin Zhou, Jun Ma, Nan Lu, Zhu Lei, Di Du, Aimin Chen

**Affiliations:** ^1^ Department of Orthopedic Trauma Surgery Shanghai Changzheng Hospital Shanghai China; ^2^ Department of Orthopaedics General Hospital of Northern Theater Command Shenyang China

**Keywords:** osteoclast, osteoporosis, P2X7, tumour necrosis factor‐α

## Abstract

The exact mechanism of tumour necrosis factor α (TNF‐α) promoting osteoclast differentiation is not completely clear. A variety of P2 purine receptor subtypes have been confirmed to be widely involved in bone metabolism. Thus, the purpose of this study was to explore whether P2 receptor is involved in the differentiation of osteoclasts. Mouse bone marrow haematopoietic stem cells (BMHSCs) were co‐cultured with TNF‐α to explore the effect of TNF‐α on osteoclast differentiation and bone resorption capacity in vitro, and changes in the P2 receptor were detected at the same time. The P2 receptor was silenced and overexpressed to explore the effect on differentiation of BMHSCs into osteoclasts. In an in vivo experiment, the animal model of PMOP was established in ovariectomized mice, and anti‐TNF‐α intervention was used to detect the ability of BMHCs to differentiate into osteoclasts as well as the expression of the P2 receptor. It was confirmed in vitro that TNF‐α at a concentration of 20 ng/mL up‐regulated the P2X7 receptor of BMHSCs through the PI3k/Akt signalling pathway, promoted BMHSCs to differentiate into a large number of osteoclasts and enhanced bone resorption. In vivo experiments showed that more P2X7 receptor positive osteoclasts were produced in postmenopausal osteoporotic mice. Anti‐TNF‐α could significantly delay the progression of PMOP by inhibiting the production of osteoclasts. Overall, our results revealed a novel function of the P2X7 receptor and suggested that suppressing the P2X7 receptor may be an effective strategy to delay bone formation in oestrogen deficiency‐induced osteoporosis.

## 1 INTRODUCTION

Osteoporosis is a common outcome of the ageing population, with more than 200 million patients in the world, which is the third highest incidence of a disease in the elderly. Postmenopausal women experience bone loss and further form osteoporosis, referred to as postmenopausal osteoporosis (PMOP).^1^ Early research has shown that the serum inflammatory indexes of women with PMOP remain high. Patients with inflammatory diseases also had high levels of inflammatory factors, and the prevalence rate of osteoporosis was 15 times higher than that in people of the same age without inflammatory diseases.^2^ Therefore, inflammatory factors are essential in the occurrence and development of osteoporosis. After menopause, the decrease of oestrogen and increase of inflammatory factors in middle‐aged and elderly women disrupt the dynamic balance between osteoblasts and osteoclasts, leading to osteogenic reduction, bone loss and osteoporosis.

Numerous studies have shown that the decrease of postmenopausal oestrogen levels can increase self‐inflammatory cytokines and inflammatory bodies, including IL‐1, IL‐6 and tumour necrosis factor α (TNF‐α), thus promoting an increased number of osteoclasts. Among these inflammatory factors, the level of TNF‐α increased most significantly, and in vitro experiments showed that IL‐1 and IL‐6 alone could not increase the number of differentiated osteoclasts.^3^, ^4^, ^5^

Purine energy signals are associated with P2 receptors and have been confirmed to regulate the balance between bone resorption and formation. Genetic studies have found a correlation between P2 polymorphism and increased risk of osteoporosis. For example, animals with P2 gene knockout develop bone deformities.^6^ There are two types of P2 receptors: P2X and P2Y. Currently, seven subtypes of P2X (P2X1, 2, 3, 4, 5, 6, 7) and eight P2Y receptor subtypes (P2Y1, 2, 4, 6, 11, 12, 13, 14) have been found. The expression of several P2X and P2Y receptor subtypes has been determined in different types of osteocytes. Furthermore, the seven subtypes of P2X and four of the subtypes of P2Y (1, 2, 4, 6) are closely related to the regulation of calcium ion in osteoclasts and osteoblasts.

TNF‐α promotes the progress of osteoporosis, and the P2 receptor also increases as osteoclasts increase. Therefore, it is hypothesized that TNF‐α can affect the P2 receptor, thereby increasing osteoclasts and aggravating the progression of osteoporosis. Previous studies have found that TNF‐α increases the number of P2Y2 receptors and suppresses osteoblast differentiation. However, the effect of the P2 receptor on osteoclasts affected by TNF‐α remains unclear.

Another potential mechanism of osteoclast differentiation could be confirmed by studying the effects of TNF‐α on P2 receptor, osteoclast differentiation and bone resorption of mouse bone marrow haematopoietic stem cells (BMHSCs). This study mainly focuses on the effect of TNF‐α on BMHSCs. We found that TNF‐α significantly promoted the differentiation of BMHSCs into osteoclasts and up‐regulated the expression of specific purine receptors. Inhibition of this receptor significantly inhibited the effect of TNF‐α on BMHSCs. In addition, the results suggest that TNF‐α up‐regulates P2 receptor by activating the Akt signalling pathway, which affects the regulation of calcium ion in BMHSCs, and further affects the differentiation of osteoclasts, enhances bone resorption and aggravates the progress of osteoporosis. The results of this study provide a potential mechanism for the occurrence and development of osteoporosis and provide basic research results for future therapeutic targets.

## 2 MATERIALS AND METHODS

### 2.1 In vivo experiments

Animal experiments were carried out in accordance with the guidelines of the Medical Ethics Committee of Shanghai Changzheng Hospital. Eight‐week‐old female C57BL/6J mice were randomly divided into three groups (n = 9/group). The sample number was based on a preliminary experiment (90% confidence interval, 5% type I error risk and 10% type II error risk): control group (no operation, but exposure of bilateral ovaries, retained intact and injection of 0.1 mL saline/3 d); model group (bilateral ovariectomy, and injection of 0.1 ml saline/3 d); and experimental group (bilateral ovariectomy, 0.1 mL injection of anti‐TNF‐α, 100 mg/kg, R&D Systems). Eight weeks later, three mice were randomly selected from each group for the following experiments: (a) serum, separated by centrifugation from blood taken from the eyeball, was detected and analysed using an ELISA (Bio Legend); (b) microscopic CT scanning was performed on bilateral femur and tartrate‐resistant acid phosphatase (TRAP) staining (TRAP kit, Sigma), HE staining and immunohistochemical staining were performed on bilateral tibia. The remaining mice in each group (n = 5) were used for cell experiments.

### 2.2 In vitro experiments

BMHSCs were isolated from the bone marrow of femur and tibia of 8‐week‐old female C57BL/6 mice and cultured in 10 mL medium (α‐MEM + 10% foetal bovine serum and 100 μg/mL penicillin/streptomycin). After culturing in a cell incubator at 37°C and 5% CO_2_ for 24 hours, the supernatant was centrifuged (3000g/min, 15 minutes) to obtain osteoclast precursor cell precipitate. Then the cells were treated with M‐CSF (30 ng/mL) and RANKL (50 ng/mL). The optimal stimulating concentration of TNF‐α was 20 ng/mL, and the maturation time of osteoclasts was 5.5 ± 1.1 days.

Osteoclast progenitor cells were inoculated into 6‐well tissue culture plates at a density of 3 × 10^4^/mL. When the cells grew to about 50% confluence, they were infected for 24 hours by adding P2X7 receptor shRNA lentivirus particles (Santa Cruz Biotechnology) to the culture medium. The control group cells were sham infected by adding shRNA‐P2X7‐negative‐control (shRNA NC) for 24 hours. Different samples were collected after culturing for different times to run qRT‐PCR detection, Western blot, TRAP staining (TRAP kit, Sigma), cyclopeptide staining, immunofluorescence staining and absorption indentation test of bone slices (cultured continuously for 14 days). TRAP^+^ cells with more than three nuclei were counted as osteoclasts. For the in vitro work, the experiment was repeated with three independent biological samples, each done in triplicate.

#### 2.2.1 Detection of cell proliferation and cytotoxicity using cell counting kit‐8

The cells were cultured in different media with a density of 10^3^‐10^4^ cells/well in a 96‐well plate in an incubator. After 24 hours, 10 μL Cell Counting Kit‐8 (CCK‐8 kit, MCE) solution was added to each well for another reaction of 4 hours. Then to ensure the liquid colour in the well was evenly mixed, the plate was gently shaken for 1 minute. The absorbance at 450 nm was measured using the Infinite F200/M200 (Tecan, Switzerland) enzyme labelling instrument.

#### 2.2.2 qRT‐PCR

The cells were lysed with Trizol (Invitrogen), and RNA from the cells was purified by dissolution of chloroform, low temperature centrifugation, precipitation of isopropanol and alcohol washes. Using a SYBR Premix kit (Takara) and the manufacturer's instructions, RNA reverse transcription was performed to synthesize DNA, and amplification was performed using an ABI 7500 Real‐Time System (ABI) by means of incorporation of SYBR Green fluorescent dye as described in the protocols. Beta actin was used as a reference gene to detect the changes in cellular P2 receptors and osteoclast markers using the 2^−ΔΔCt^ method.

#### 2.2.3 Osteoclast ability test: bone sag test

In order to investigate the effect of TNF‐α stimulation on the function of osteoclasts by increasing P2X7 receptor, osteoclasts were divided into three groups according to the density of 3 × 10^4^/mL and continuously cultured (calf femur, bone biomimetic synthetic coating plate, COSMO BIO). Fourteen days later, the surface indentation area of bone slices was observed and measured by TRAP staining and fluorescence microscopy, and the area of bone indentation was quantified.

#### 2.2.4 Immunofluorescence

In order to clarify the effect of TNF‐α on osteoclast differentiation and P2X7 receptor, immunofluorescence was used to detect the number of osteoclasts and P2X7 receptor of osteoclast progenitor cells in the presence or absence of TNF‐α. A total of 10^3^ cells in each group were cultured for 5 days, then fixed with 4% neutral formaldehyde for 20 minutes and rinsed three times with PBS. Immunofluorescence staining was performed using DAPI, phalloidin staining (Alexa Fluor 594 Phalloidin) and receptor antibody immunofluorescence staining.

#### 2.2.5 Western blot

Protein samples of cells or tissues were separated with SDS‐PAGE (7.5%‐10% polyacrylamide gel), and protein imprinting was polymerized on a vinylidene fluoride membrane (0.45 mol/L, Millipore) via an electroporation membrane. Then 5% skimmed milk powder was used to seal the membrane in TBS brine for 1 hour. The membrane was then incubated with a specific antibody (1:400) at 4°C for 16 hours (Santa Cruz Biotechnology), followed by a second antibody (1:5000) at room temperature for 4 hours (Santa Cruz Biotechnology), The antigen‐antibody complex was observed using an enhanced chemiluminescence analysis (Thermo Scientific).

The effects of TNF‐α on NF‐kB, MAPK and AKT pathways in osteoclast progenitor cells were detected by Western blot. Osteoclast progenitor cells (10^5^ cells/well) were inoculated into 6‐well plates and divided into two groups: RANKL‐treated group and Rankl + TNF‐α‐treated group (20 ng/mL). The cells were detected by Western blotting at 5, 15, 15, 30, 45, 55 and 60 minutes, and the phosphorylation of Akt, PI3k, MAPK, c‐fos, IKB and NF‐kB was observed. Through different stimulation culture methods, the expression level of TRAP, Cathepsin K (CTSK), MMP‐9, C‐Scr, and markers related to osteoclast production were detected by Western blotting. The primary antibody included mouse‐derived anti‐actin, anti‐Akt, PI3k, MAPK, c‐fos, IKB, NF‐kB phosphorylated antibodies, cathepsin K, C‐Scr, Trap, NFATc1 and other marker protein antibodies. The secondary antibody was anti‐rabbit IgG.

### 2.3 Statistical analyses

Data were analysed using SPSS software (SPSS 11.5). Two‐tailed unpaired one‐way ANOVA was used to compare between more than two groups. Bars are the mean ± SD of countable data of biological samples. Results were considered statistically significant if *P* ≤ .05.

## 3 RESULTS

### 3.1 TNF‐α promotes the differentiation of BMHSCs into osteoclasts in vitro

**Figure 1:**
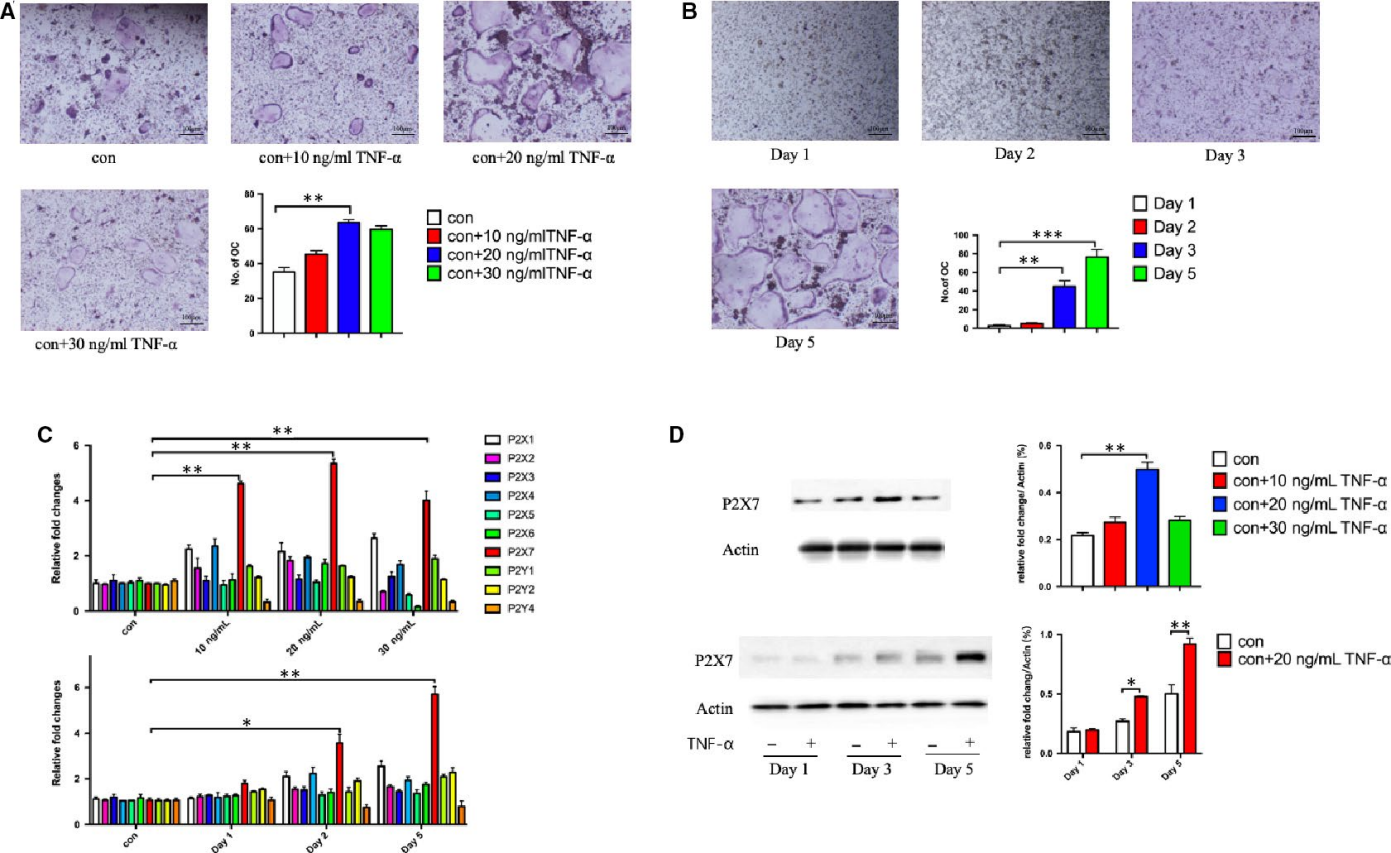
The results of in vitro experiments confirmed that TNF‐α at 20 ng/mL significantly up‐regulated the expression of P2X7 receptor protein of BMHSCs and promoted their differentiation into more osteoclasts. A, BMHSCs were stimulated by different concentrations of TNF‐α to differentiate into osteoclasts, and then TRAP staining was used for statistical comparison (n = 5, scale bars were 100 μm). B, BMHSCs were stimulated by a 20 ng/mL concentration of TNF‐α to differentiate into osteoclasts, and then stained with TRAP for statistical comparison (n = 5, scale bars were 100 μm). C, The mRNA of P2 receptors in BMHSCs were detected by qRT‐PCR after stimulation by different concentrations of TNF‐α for different times (n = 5). D, P2X7 receptor protein of BMHSCs was detected by Western blot after stimulation by different concentrations of TNF‐α for different times (n = 5). *P < .05, **P < .01, ***P < .001

In order to explore the effect of TNF‐α on BMHSC differentiation, mouse BMHSCs were isolated and cultured with different concentrations of TNF‐α. Then TRAP staining was performed to count the number of differentiated osteoclasts to find the most significant concentration (Figure 1A). After culturing with the most significant stimulation concentration, cells were stained with TRAP at different times, and the number of differentiated osteoclasts was counted to find the most suitable time (Figure 1B). Under the 20 ng/mL TNF‐α concentration, BMHSCs differentiated into more osteoclasts after 5 days of culture, and the difference was statistically significant (*P* < .05). Based on qRT‐PCR detection, the P2 receptors were shown to increase to different degrees in the process of osteoclast differentiation of BMHSCs stimulated by different concentrations of TNF‐α; the largest increases occurred with P2X7 receptor (Figure 1C). It was further verified by Western blot that the expression of P2X7 protein in BMHSCs was significantly up‐regulated after 5 days of stimulation with 20 ng/mL of TNF‐α (Figure 1D). The changes in P2X7 receptor expression are related to the number of differentiated BMHSCs, that is, with increased P2X7 receptor protein expression, the number of osteoclasts increased.

### 3.2 TNF‐α promoted osteoclast differentiation and bone resorption by up‐regulating P2X7 receptor in vitro

**Figure 2:**
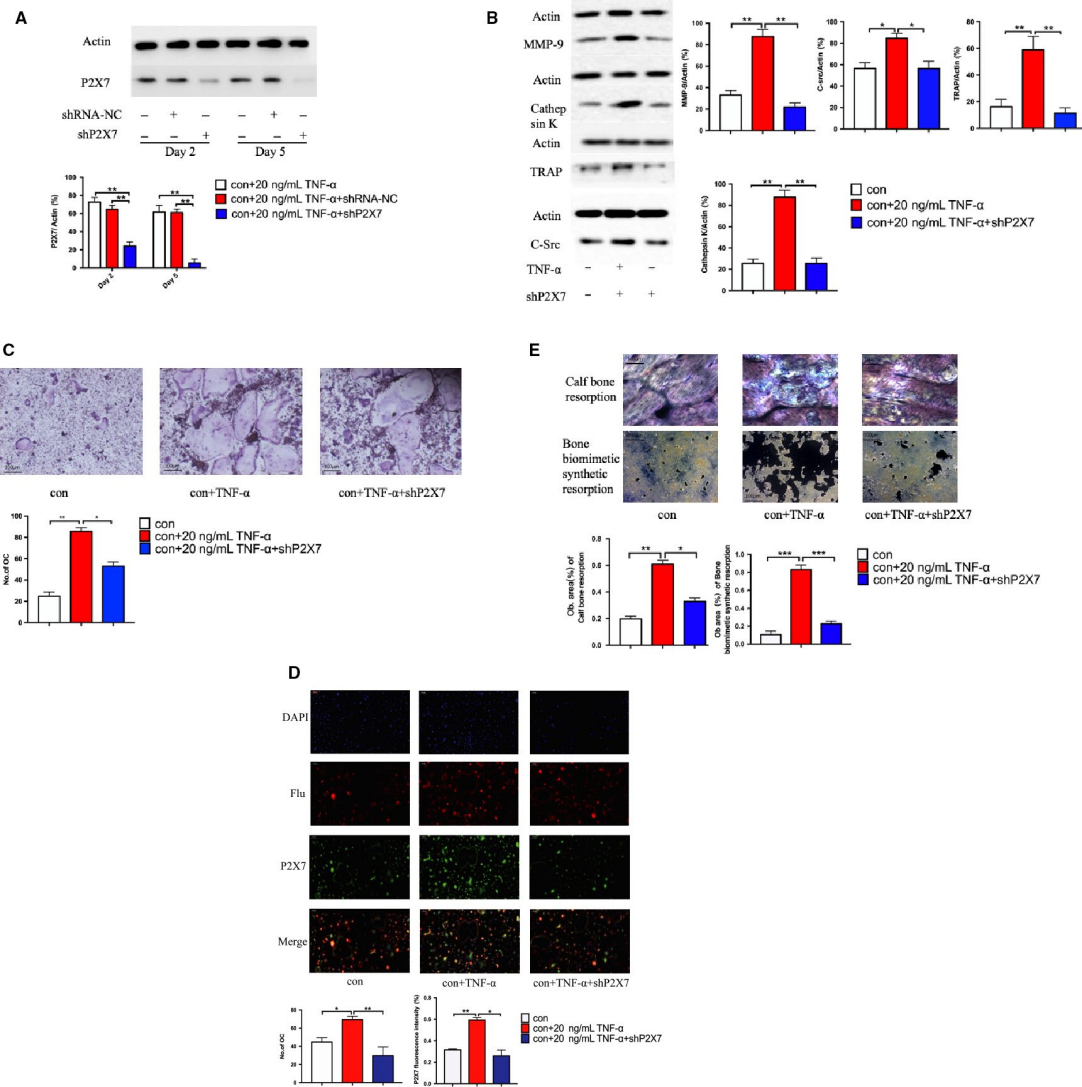
The results of an in vitro experiment showed that TNF‐α was involved in the differentiation of BMHSCs into osteoclasts by up‐regulating the P2X7 receptor. A, The silencing effect of transfected shRNA‐P2X7 was detected by Western blot (n = 5). B, The specific proteins related to osteoclast differentiation expressed by BMHSCs transfected with shRNA‐P2X7 were detected by Western blot, including MMP‐9, Cathepsin K, TRAP, and C‐Src (n = 5). C, The differentiation of osteoclasts from BMHSCs transfected with shRNA‐P2X7 was detected by TRAP staining and statistically analysed (n = 5, scale bars were 100 μm). D, After BMHSCs differentiated into osteoclasts, they were stained with cyclopeptide and immunofluorescence, and the number of osteoclasts and P2X7 receptors were counted (P2X7‐Green) (n = 5, scale bars were 100 μm). E, The experiment of osteoclast bone resorption was carried out with bovine bone slices and a biomimetic bone coated petri dish (two different methods). And the bone resorption areas of the two materials were analysed quantitatively (n = 5, scale bars were 100 μm). *P < .05, **P < .01, ***P < .001

In order to observe the effect of the P2X7 receptor on osteoclast differentiation of BMHSCs, we first used shRNA‐P2X7 to silence the mRNA of the receptor (overexpression of the receptor did not increase the number of differentiated cells). Western blot analysis showed that deletion of shRNA‐P2X7 receptor was effective at different time points (2 and 5 days) (Figure 2A). BMHSCs with mRNA of silenced P2X7 receptor were cultured in osteoclast differentiation medium for 5 days, and then the osteoclast‐specific genes, MMP‐9, TRAP, Cathepsin K (CTSK) and C‐src, were detected by qRT‐PCR and Western blot. The results showed that the expression levels of MMP‐9, TRAP, Cathepsin K (CTSK) and C‐src in BMHSCs transfected with shRNA‐P2X7 were significantly decreased (Figure 2B). In addition, shRNA of P2X7 receptor inhibited the osteoclast differentiation potential of BMHSCs, as shown by TRAP staining (Figure 2C). In addition, specific immunohistochemical staining showed that TNF‐α significantly increased the number of P2X7 receptors in osteoclasts and promoted osteoclast differentiation (Figure 2D). The bone resorption indentation test (Figure 2E) showed that TNF‐α promoted osteoclast differentiation of BMHSCs and increased the bone resorption capacity of osteoclasts, while inhibition of the P2X7 receptor significantly reduced the effect of TNF‐α on BMHSCs. These results strongly suggest that P2X7 receptors were involved in the differentiation of BMHSC osteoclasts. Inhibition of P2X7 receptor protein expression can significantly reduce the number of differentiated osteoclasts and inhibit osteoclast bone resorption.

### 3.3 TNF‐α up‐regulated the expression of P2X7 through the PI3k/Akt signalling pathway promoting osteoclast differentiation induced by RANKL

**Figure 3:**
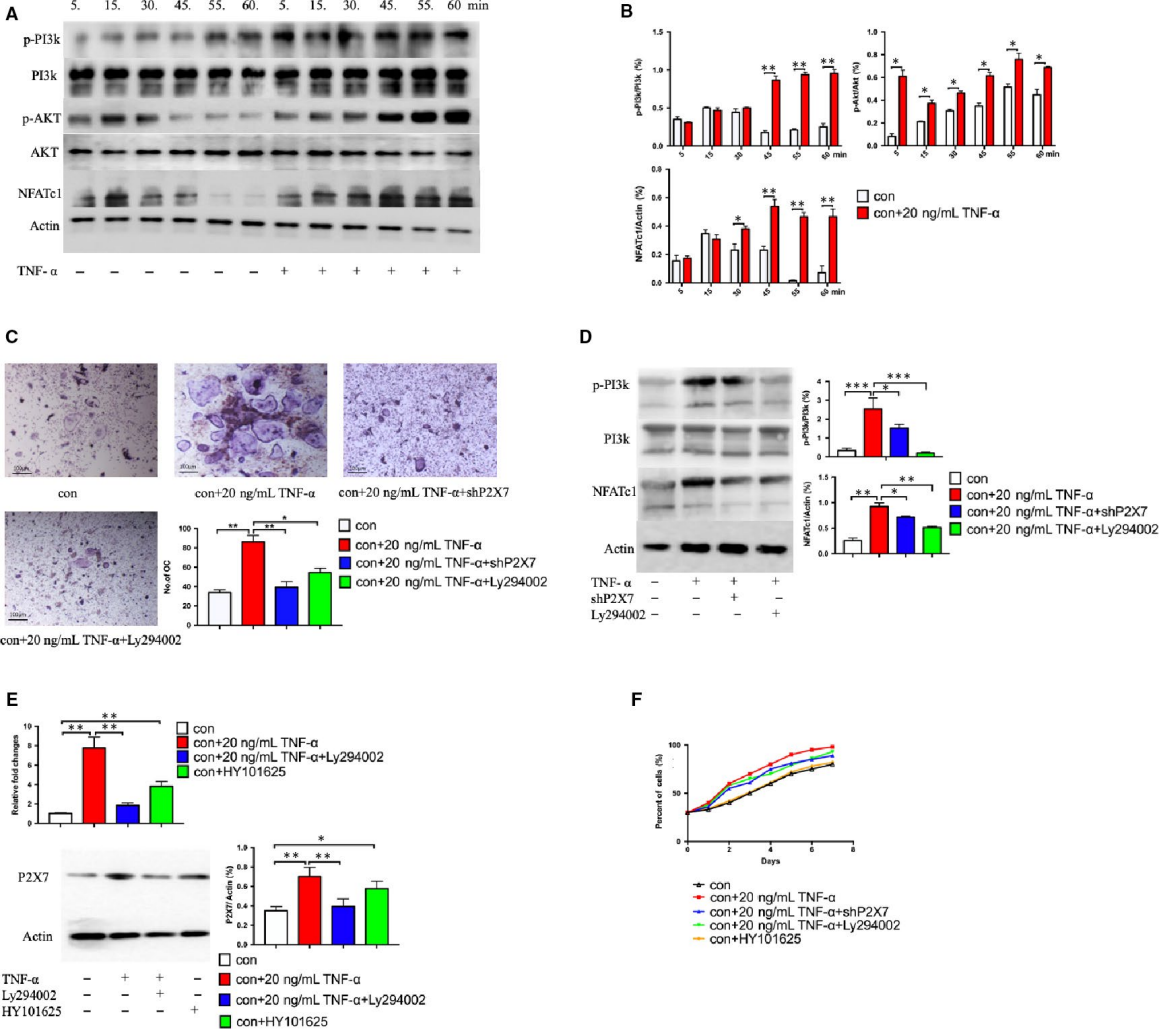
TNF‐α up‐regulates the expression of P2X7 through the PI3k/Akt signalling pathway, thus promoting osteoclast differentiation induced by RANKL. A, The transient phosphorylation of the signalling pathway proteins in BMHSCs stimulated by TNF‐α was detected by Western blot (n = 5). B, The quantitative data of the phosphorylation change of instantaneous signal protein in (A) were presented (n = 5). C, BMHSCs were stimulated by Ly294002, an inhibitor of the PI3k/Akt signal pathway, and the differentiated osteoclasts were stained with TRAP (n = 5, scale bars were 100 μm). D, Changes of PI3k/Akt protein phosphorylation and NFATc1 protein expression in BMHSCs stimulated by Ly294002 were detected by Western blot and analysed statistically (n = 5). E, Changes of P2X7 receptor mRNA and protein expression in BMHSCs stimulated by inhibitor Ly294002/activator HY101625 were detected by Western blot and analysed statistically (n = 5). F, The change of number of BMHSCs under the stimulation of different reagents was detected by CCK8 (n = 5). *P < .05, **P < .01, ***P < .001

In addition to the PI3k/Akt signalling pathway, activation of MAPKs and the NF‐kB pathway plays an important role in differentiation of osteoclasts. To evaluate the effects of TNF‐α on the PI3k/Akt, NF‐kB, and MAPK pathways after incubation with RANKL, we firstly examined the phosphorylation of major subfamilies of the signalling pathways, such as phosphorylated PI3k (p‐PI3k), Akt (p‐Akt), ERK (p‐ERK) and C‐fos (p‐c‐fos) by Western blot analysis. Among the major subfamilies, p‐ERK and p‐c‐fos levels did not change significantly, but p‐PI3k and p‐Akt showed a significant increase upon TNF‐α stimulation (Figure 3A,B). NFATc1, the osteoclastogenesis‐related marker, was increased at the same time. Secondly, when PI3k was specifically inhibited by Ly294002 (Apex BIO), it significantly weakened the effect of TNF‐α on the differentiation of BMHSCs, including the number of differentiated osteoclasts (Figure 3C) and osteoclast differentiation‐related protein, NFATc1 (Figure 3D). Thirdly, when the PI3k/Akt signalling pathway was activated by HY101625 (MCE), the expression of P2X7 protein increased; on the contrary, when the signalling pathway was inhibited by Ly294002, the expression of P2X7 protein decreased accordingly (Figure 3E). In addition, the results of CCK‐8 showed that there was no statistical difference in the effect of different reagents on the number of osteoclasts (Figure 3F).

### 3.4 TNF‐α up‐regulated the expression of P2X7 in PMOP

In order to clarify the effect of TNF‐α on PMOP osteoclasts, we performed ovariectomized (OVX) or sham operation in 8‐week‐old female mice. Three days later, OVX mice were treated with anti‐TNF‐α or saline, and the sham operation group was treated with the same dosage of physiological saline twice a week for 8 weeks.

**Figure 4:**
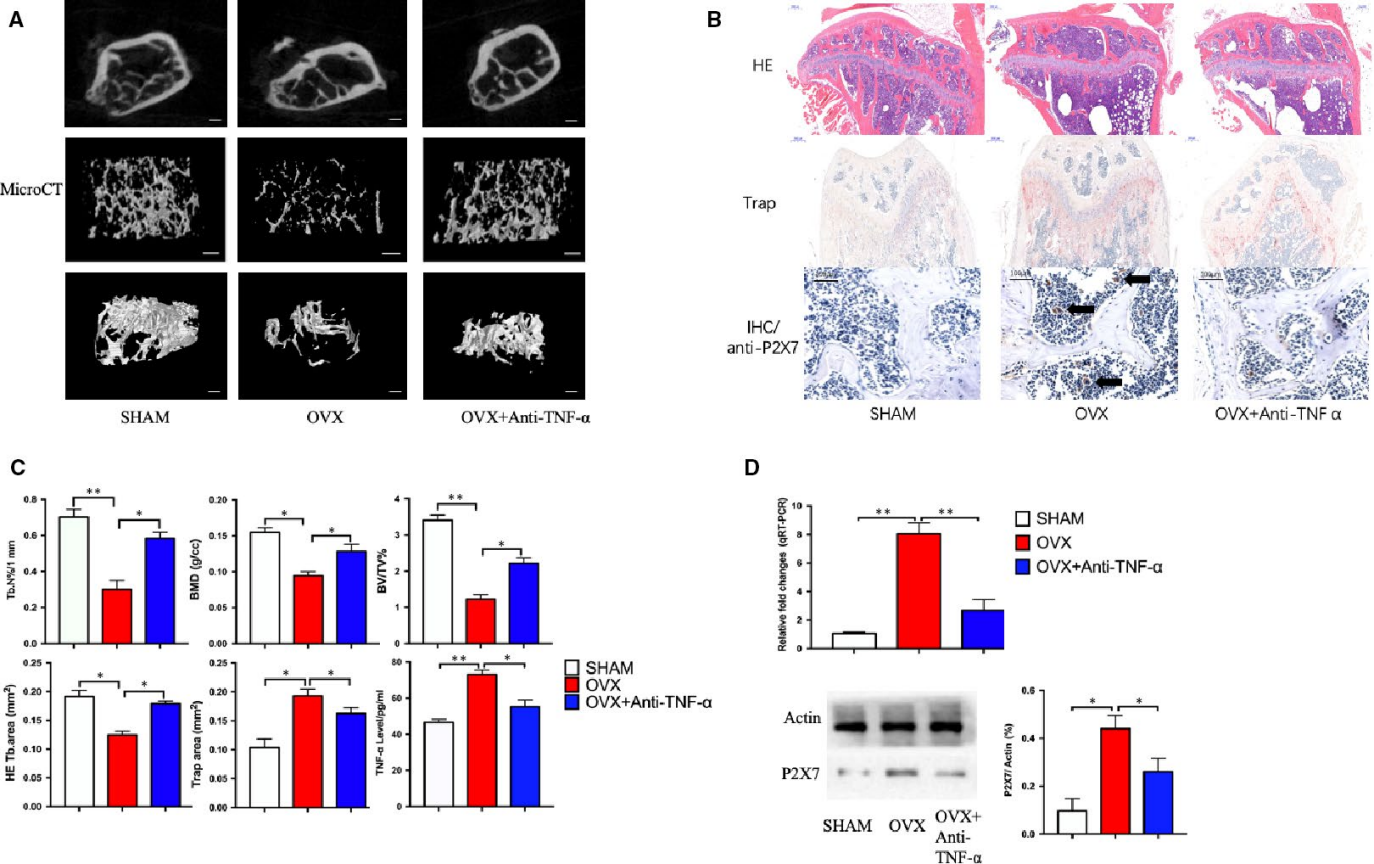
With increased serum TNF‐α in ovariectomized mice, the number of the osteoclasts increased, while bone trabeculae, bone mineral density and bone content decreased, compared to the SHAM mice. At the same time, the expression of P2X7 in BMHSCs was up‐regulated. Anti‐TNF‐α can effectively inhibit the effect of TNF‐α factor on BMHSCs. A, Randomly selected specimens were scanned by Micro‐CT and reconstructed. The image shows three groups of typical images (n = 4, scale bars were 50 μm). B, Three groups of specimens were stained with HE (scale bars were 200 μm), TRAP (scale bars were 200 μm) and P2X7 protein fluorescence staining (IHC, arrows show a large number of P2X7 protein positive osteoclasts in the OVX group, scale bars were 100 μm). The image shows three groups of typical images (n = 4). C, Quantitative data analysis of Micro‐CT scan reconstruction (Tb.N, BMD, BV/TV), HE staining (HE Tb.N‐area) and TRAP staining (TRAP area) was calculated statistically, and the content of TNF‐α in serum (TNF‐α level) of three groups of mice was detected by ELISA (n = 4). D, The P2X7 receptor mRNA was detected by qRT‐PCR (relative fold changes) and protein expression in bone tissue was detected by Western blot (n = 4). *P < .05, **P < .01, ***P < .001

The results of Micro‐CT analysis showed that compared with the sham‐operated control group, the trabecular structure of OVX mice treated with anti‐TNF‐α was significantly improved compared with the untreated group (Figure 4A). The results of HE staining (bone trabecular structure) were similar (Figure 4B). The results of TRAP staining showed that anti‐TNF‐α treatment significantly inhibited the formation of osteoclasts in OVX mice. Immunofluorescence results showed that a large number of P2X7 positive osteoclasts were found in the femur of OVX mice (IHC in Figure 4B). Statistics from Micro‐CT scans showed that the BMD and BV/TV of femur in OVX mice decreased, and Tb. N% increased (Figure 4C). In order to confirm that the mouse models used in this study were reasonable and reliable, we measured the level of TNF‐α in the serum of mouse models by ELISA. The results showed that the level of TNF‐α was significantly increased in OVX mice and significantly attenuated in OVX + anti‐TNF‐α mice (Figure 4C). In addition, by detecting the osteoclast progenitor cells formed by BMHSCs in the three groups, it was found that the P2X7 receptor in OVX cells increased significantly (Figure 4D).

## 4 DISCUSSION

Destruction of the dynamic balance between osteoblasts and osteoclasts is an important pathological mechanism of PMOP. Through detection of the purine receptor gene in postmenopausal women, some scholars have found a correlation between purine receptor polymorphism and bone mineral density.^7^ The results of the experiments show that TNF‐α can inhibit bone formation by up‐regulating P2Y2 receptor to inhibit the differentiation of bone marrow mesenchymal stem cells into osteocytes.^2^ In this study, TNF‐α was found to promote BMHSCs to differentiate into osteoclasts by up‐regulating P2X7 receptors, thus promoting bone destruction and absorption.

Some studies have shown that low concentrations of TNF‐α (1‐5 ng/mL) promote osteoblast differentiation through the NF‐κB signal pathway,^8^ while inhibiting osteoclast differentiation.^9^ Clinically, the serum concentration of TNF‐α in patients with inflammatory disease is 3‐10 times higher than the normal level, and they are 15 times more likely to suffer from osteoporosis.^10^ In addition, serum TNF‐α also increases significantly in postmenopausal women. Therefore, a high concentration of TNF‐α can promote the differentiation of osteoclasts. In this experiment, a higher stimulating concentration (20 ng/mL) significantly promoted the differentiation of BMHSCs into more osteoclasts, which is consistent with the findings of clinical practice. The results of this experiment will help researchers better understand the different roles and mechanisms of TNF‐α in osteoclasts, to provide a basis for clinical treatment in the future.

Previous studies showed that the P2 receptor is related to inflammation and cellular immune response, as well as energy metabolism.^3^, ^11^ Previous studies have shown that P2X5, P2X7, P2Y1 and P2Y2 receptors are essential for osteocyte differentiation and proliferation.^11^, ^12^ Moreover, it has been confirmed that a variety of purine receptors are related to calcium channels and are closely related to the differentiation and proliferation of osteoblasts, osteoclasts and their progenitors.^13^ Researchers have found that mice with P2X7 receptor knockout have reduced periosteal formation and increased trabecular bone resorption.^14^ The activation of P2X7 receptor increases the production of lipophosphatidic acid (LPA) and prostaglandin E2, leading to osteoblast apoptosis by affecting the Akt/GSK3β/β‐catenin signal pathway.^15^ However, the relationship between purine receptors and TNF‐α has not been clearly confirmed. This is the first time that TNF‐α stimulation has been shown to significantly up‐regulate the P2X7 receptor of BMHSCs in PMOP. TNF‐α up‐regulates P2X7 receptor through the PI3k/Akt signal pathway, which regulates intracellular calcium concentration and secondary messengers, and further regulates transcription factors into the nucleus, and promotes BMHSCs to differentiate into osteoclasts.^16^, ^17^

However, the relationship between purine receptors and osteoclasts has not been clearly confirmed.^18^ Studies have shown that P2X7 receptors produced on apoptotic osteocytes are compulsory for adjacent osteocytes to produce RANKL.^19^ Although the P2X7 receptor is not necessary for osteoclast fusion, its presence and increase promote the increase of RANKL and further promote the differentiation and formation of osteoclasts.^20^ The results of this study demonstrate that the P2X7 receptor promotes BMHSCs to differentiate into more functional osteoclasts. Further studies have found that the P2X7 receptor could promote osteoclast formation by inhibiting TRAF6 and activating the PI3k/Akt pathway,^21^ which still need further in vivo experiments to confirm.

Previous studies have suggested that purine receptors are affected by ATP,^22^ which in turn affects cellular calcium channels and downstream signal pathways.^23^ This is consistent with the results of our experiment. After inhibiting the PI3k/Akt pathway, the effect of TNF‐α on osteoclasts is almost the same as knockout of the P2X7 receptor gene. The osteoclast differentiation pathway is mainly mediated by C‐Src‐mediated PI3k/Akt and TRAF6‐mediated IκB/NF‐κB, MAPK/c‐fos.^24^ Previous studies have suggested that under the influence of ATP, purine receptors will promote the opening of mechanical and L‐type calcium channels, and influx of calcium ions,^25^ which will affect some signalling pathways.^9^ However, this is an instantaneous effect, and confirming it requires long‐term experiments on the signalling pathway. The results of this experiment show that TNF‐α can up‐regulate the P2X7 receptor by the PI3k/Akt pathway.

For postmenopausal women, oestrogen deficiency can induce T cells to secrete more TNF‐α. Both in vivo and in vitro experiments showed that TNF‐α promoted the differentiation of osteoclasts by up‐regulating the P2X7 receptor of BMHSCs. Therefore, the P2X7 receptor is a potential effective therapeutic target against PMOP and is of research significance. Therefore, to some extent for PMOP, inhibiting the P2X7 receptor of BMHSCs can inhibit the differentiation of osteoclasts and delay bone loss. However, the effect of the P2X7 receptor on the immune response and inflammation of BMHSCs needs further research.

This study focused on osteoclasts and their progenitor‐hematopoietic stem cells, and simply but systematically studied the effects of postmenopausal tumour necrosis factor‐α on their differentiation and bone resorption and identified an important role and mechanism of the P2X7 purine receptor.

In this study, we found that TNF‐α significantly up‐regulates the P2X7 receptor of BMHSCs by activating the AKT signalling pathway, regulating calcium metabolism, promoting BMHSCs to differentiate into more osteoclasts, affecting the dynamic balance between osteoblasts and osteoclasts, and aggravating the progress of osteoporosis. Therefore, inhibiting P2X7 receptor significantly reduces the effect of TNF‐α on BMHSCs and reduces the formation of osteoclasts. This study found that P2X7 was a potential target for anti‐osteoporosis therapy and provided a basis for future research.

## CONFLICT OF INTEREST

The authors declare no financial conflict of interest with each other involving this study.

## AUTHOR CONTRIBUTION

**Jiajia Lu:** Data curation (lead); Formal analysis (lead); Investigation (lead); Methodology (lead); Resources (lead); Software (lead); Writing‐original draft (lead). **Zhibin Zhou:** Conceptualization (supporting); Data curation (equal); Formal analysis (supporting); Investigation (equal); Project administration (supporting); Supervision (supporting); Visualization (supporting); Writing‐review & editing (supporting). **Jun Ma:** Conceptualization (supporting); Data curation (supporting); Investigation (supporting); Project administration (equal); Validation (supporting); Writing‐review & editing (supporting). **Nan Lu:** Data curation (supporting); Project administration (supporting); Supervision (supporting). **Lei Zhu**: Conceptualization (supporting); FormalAnalysis (supporting); Methodology (supporting); WritingReviewEditing (supporting). **Di Du:** Conceptualization (supporting); Formal analysis (supporting); Methodology (supporting); Project administration (supporting); Resources (supporting); Supervision (supporting); Writing‐review & editing (supporting). **Aimin Chen:** Conceptualization (lead); Supervision (lead).

## CONSENT FOR PUBLICATION

My manuscript does not contain data from any individual person.

## Data Availability

The datasets during and/or analysed during the current study available from the corresponding author on reasonable request.

## References

[1] Chen X , Zhi X , Pan P , et al. Matrine prevents bone loss in ovariectomized mice by inhibiting RANKL‐induced osteoclastogenesis. FASEB J. 2017;31:4855‐4865. 28739641 10.1096/fj.201700316R PMC5636701

[2] Du D , Zhou Z , Zhu L , et al. TNF‐α suppresses osteogenic differentiation of MSCs by accelerating P2Y2 receptor in estrogen‐deficiency induced osteoporosis. Bone. 2018;117:161‐170. 30236554 10.1016/j.bone.2018.09.012

[3] Fuller K , Kirstein B , Chambers TJ . Regulation and enzymatic basis of bone resorption by human osteoclasts. Clin Sci. 2007;112:567‐575. 10.1042/CS20060274 17241109

[4] O'Donnell S , O'Morain C . Review article: use of antitumour necrosis factor therapy in inflammatory bowel disease during pregnancy and conception. Aliment Pharmacol Ther. 2008;27:885‐894. 18284649 10.1111/j.1365-2036.2008.03648.x

[5] Yang N , Wang G , Hu C , et al. Tumor necrosis factor alpha suppresses the mesenchymal stem cell osteogenesis promoter miR‐21 in estrogen deficiency‐induced osteoporosis. J Bone Miner Res. 2013;28:559‐573. 23074166 10.1002/jbmr.1798

[6] Jorgensen NR , Henriksen Z , Sorensen OH , Eriksen EF , Civitelli R , Steinberg TH . Intercellular calcium signaling occurs between human osteoblasts and osteoclasts and requires activation of osteoclast P2X7 receptors. J Biol Chem. 2002;277:7574‐7580. 11756404 10.1074/jbc.M104608200

[7] Xiao W , Gong C , Liu X , et al. Association of P2X7R gene with serum lipid profiles in Chinese postmenopausal women with osteoporosis. Climacteric. 2019;22:498‐506. 31079509 10.1080/13697137.2019.1604654

[8] Kulkarni RN , Voglewede PA , Liu D . Mechanical vibration inhibits osteoclast formation by reducing DC‐STAMP receptor expression in osteoclast precursor cells. Bone. 2013;57:493‐498. 23994170 10.1016/j.bone.2013.08.020 PMC4589847

[9] Sang C , Zhang Y , Chen F , et al. Tumor necrosis factor alpha suppresses osteogenic differentiation of MSCs by inhibiting semaphorin 3B via Wnt/beta‐catenin signaling in estrogen‐deficiency induced osteoporosis. Bone. 2016;84:78‐87. 26723579 10.1016/j.bone.2015.12.012

[10] Maria S , Swanson MH , Enderby LT , et al. Melatonin‐micronutrients Osteopenia Treatment Study (MOTS): a translational study assessing melatonin, strontium (citrate), vitamin D3 and vitamin K2 (MK7) on bone density, bone marker turnover and health related quality of life in postmenopausal osteopenic women following a one‐year double‐blind RCT and on osteoblast‐osteoclast co‐cultures. Aging. 2017;9:256‐285. 28130552 10.18632/aging.101158 PMC5310667

[11] Muscella A , Cossa LG , Vetrugno C , Antonaci G , Marsigliante S . Inhibition of ZL55 cell proliferation by ADP via PKC‐dependent signalling pathway. J Cell Physiol. 2018;233:2526‐2536. 28777435 10.1002/jcp.26128

[12] Sekar P , Huang D‐Y , Hsieh S‐L , Chang S‐F , Lin W‐W . AMPK‐dependent and independent actions of P2X7 in regulation of mitochondrial and lysosomal functions in microglia. Cell Commun Signal. 2018;16:83‐98. 30458799 10.1186/s12964-018-0293-3 PMC6245559

[13] Agrawal A , Gartland A . P2X7 receptors: role in bone cell formation and function. J Mol Endocrinol. 2015;54:R75‐R88. 25591582 10.1530/JME-14-0226

[14] Ma Y , Zhao H , Chile C , et al. The effect of P2X7R‐mediated Ca(2+) signaling in OPG‐induced osteoclasts adhesive structure damage. Exp Cell Res. 2019;383:111555. 31415763 10.1016/j.yexcr.2019.111555

[15] Zhang Y , Li W , Liu C , et al. Electromagnetic field treatment increases purinergic receptor P2X7 expression and activates its downstream Akt/GSK3beta/beta‐catenin axis in mesenchymal stem cells under osteogenic induction. Stem Cell Res Ther. 2019;10:407. 31864409 10.1186/s13287-019-1497-1 PMC6925409

[16] Li Y , Yin C , Liu P , Li D , Lin J . Identification of a different agonist‐binding site and activation mechanism of the human P2Y1 receptor. Sci Rep. 2017;7:13764. 29062134 10.1038/s41598-017-14268-1 PMC5653743

[17] Noronha‐Matos JB , Coimbra J , Sa‐e‐Sousa A , et al. P2X7‐induced zeiosis promotes osteogenic differentiation and mineralization of postmenopausal bone marrow‐derived mesenchymal stem cells. FASEB J. 2014;28:5208‐5222. 25169056 10.1096/fj.14-257923

[18] Jørgensen NR . Role of the purinergic P2X receptors in osteoclast pathophysiology. Curr Opin Pharmacol. 2019;47:97‐101. 30954934 10.1016/j.coph.2019.02.013

[19] Cheung WY , Fritton JC , Morgan SA , et al. Pannexin‐1 and P2X7‐receptor are required for apoptotic osteocytes in fatigued bone to trigger RANKL production in neighboring bystander osteocytes. J Bone Miner Res. 2016;31:890‐899. 26553756 10.1002/jbmr.2740 PMC4915221

[20] Dharmapatni AASSK , Algate K , Coleman R , et al. Osteoclast‐Associated Receptor (OSCAR) distribution in the synovial tissues of patients with active RA and TNF‐α and RANKL regulation of expression by osteoclasts in vitro. Inflammation. 2017;40:1566‐1575. 28555364 10.1007/s10753-017-0597-2

[21] Steinberg TH , Hiken JF . P2 receptors in macrophage fusion and osteoclast formation. Purinergic Signalling. 2007;3:53‐57. 18404418 10.1007/s11302-006-9036-9 PMC2096767

[22] Ciancetta A , O'Connor RD , Paoletta S , Jacobson KA . Demystifying P2Y1 receptor ligand recognition through docking and molecular dynamics analyses. J Chem Inf Model. 2017;57:3104‐3123. 29182323 10.1021/acs.jcim.7b00528 PMC5953180

[23] Zhao R , Qiao J , Zhang X , et al. Toll‐like receptor‐mediated activation of CD39 internalization in BMDCs leads to extracellular ATP accumulation and facilitates P2X7 receptor activation. Front Immunol. 2019;10:2524–25. 31736956 10.3389/fimmu.2019.02524 PMC6834529

[24] Chen X , Zhi X , Cao L , et al. Matrine derivate MASM uncovers a novel function for ribosomal protein S5 in osteoclastogenesis and postmenopausal osteoporosis. Cell Death Dis. 2017;8:e3037. 28880271 10.1038/cddis.2017.394 PMC5636967

[25] Wang N , Agrawal A , Jørgensen NR , Gartland A . P2X7 receptor regulates osteoclast function and bone loss in a mouse model of osteoporosis. Sci Rep. 2018;8:3507‐3517. 29472582 10.1038/s41598-018-21574-9 PMC5823935

